# Expertise Moderates Incidentally Learned Associations Between Words and Images

**DOI:** 10.3389/fpsyg.2018.02085

**Published:** 2018-10-29

**Authors:** Heather Bruett, Xiaoping Fang, Deepan C. Kamaraj, Elizabeth Haley, Marc N. Coutanche

**Affiliations:** ^1^Department of Psychology, University of Pittsburgh, Pittsburgh, PA, United States; ^2^Learning Research and Development Center, University of Pittsburgh, Pittsburgh, PA, United States; ^3^Department of Rehabilitation Science and Technology, University of Pittsburgh, Pittsburgh, PA, United States; ^4^Communication Sciences and Disorders, University of Pittsburgh, Pittsburgh, PA, United States; ^5^Brain Institute, University of Pittsburgh, Pittsburgh, PA, United States

**Keywords:** expertise, incidental encoding, associations, semantic memory, schema

## Abstract

Individuals with expertise in a domain of knowledge demonstrate superior learning for information in their area of expertise, relative to non-experts. In this study, we investigated whether expertise benefits extend to learning associations between words and images that are encountered incidentally. Sport-knowledge-experts and non-sports-experts encountered previously unknown faces through a basic perceptual task. The faces were incidentally presented as candidates for a position in a sports team (a focus of knowledge for only the sports-experts) or for a job in a business (a focus of knowledge for both the sports-experts and non-sports-experts). Participants later received a series of surprise memory tests that tested: ability to recognize each face as being old, the amount of information recalled about each face, and ability to select a correct face from equally familiar alternatives. Relative to non-sports-experts, participants with superior sports expertise were able to better recall the information associated with each face and could better select associated faces from similarly familiar options for the hypothetical prospective athletes. Hypothetical job candidates were recalled and selected at similar levels of performance in both groups. The groups were similarly familiar with the images (in a yes/no recognition memory test) when the faces were prospective athletes or job candidates. These findings suggest a specific effect of expertise on associative memory between words and images, but not for individual items, supporting a dissociation in how expertise modulates the human memory system for word–image pairings.

## Introduction

Experts remember content related to their domain of expertise at a greater level than non-experts, giving them a distinct learning advantage (Sala and Gobet, 2017). Chess masters, for example, can recreate a previously seen chessboard more accurately than chess novices (Chase and Simon, 1973). This expertise advantage can be attributed to experts’ more extensive and organized prior knowledge (Sala and Gobet, 2017). Connections within neural networks of the neocortex and subcortical areas likely allow rapid learning of new information that is related to existing knowledge (McClelland, 2013). Re-encountering existing knowledge during the learning process is hypothesized to reactivate its underlying neocortical representations, which in turn facilitates the learning and integration of new related knowledge into memory (Rawson and Van Overschelde, 2008; Herzmann and Curran, 2011; van Kesteren et al., 2012). Experts, whose neural networks hold extensive representations of expertise-related knowledge, are thereby able to rapidly learn new information in their domain of expertise. In this work, we explore how expertise affects the ability to create associative memories between unrelated stimuli. In particular, the aim of this study is to investigate the impact of expertise on the incidental encoding of associations between expertise-related words and unrelated images.

Incidental encoding describes learning that occurs without an individual being aware that learning is required. This contrasts with intentional encoding, where individuals direct their attention (and possibly rehearsal) to deliberately learn new material (Reber, 1989). Research on an expert learning advantage has predominately focused on comparisons between experts and novices in intentionally encoding semantic and episodic information (often presented as facts to memorize or past experiences to remember, such as information from passages or images; Long and Prat, 2002; Brandt et al., 2005; Herzmann and Curran, 2011), or in procedural (learning of body movement sequences, such as in ice skating; Deakin and Allard, 1991) and perceptual learning (improvement of perceptual skills, such improved memory for locations of quickly presented chess pieces; Chase and Simon, 1973). Fewer studies, however, have examined incidental encoding of semantic information (though see Hughson and Boakes, 2002; Rawson and Van Overschelde, 2008). The scarcity of studies exploring incidental encoding and expertise is important because incidental and intentional encoding are processed differently. This dissociation between incidental and intentional encoding has been shown through a variety of behavioral and neural measures (Saltzman and Atkinson, 1954; Rüsseler et al., 2003; Sharon et al., 2011; Coutanche and Thompson-Schill, 2014). One study, for example, found separate event-related potential (ERP) components related to each type of learning during a serial reaction time task (Rüsseler et al., 2003) and another has shown differences in the functional magnetic resonance imaging (fMRI) blood–oxygen-level dependent response between word–image associations learned incidentally (via fast mapping) and those learned intentionally (Merhav et al., 2015). The neural distinction between intentional and incidental encoding is perhaps most evident from studies with amnesic patients who can show successful incidental encoding of new knowledge, without an ability to learn the same information when it is presented intentionally (Sharon et al., 2011). The extent to which these types of learning differ varies, however, with some examples of the two producing similar performance at test (e.g., Utochkin and Wolfe, 2018), making it uncertain whether a given effect found for intentional learning will also be found for incidental encoding. There is evidence from intentional encoding studies that experts recall (or remember specific details about) more associative information within their area of expertise, but studies fail to show this effect on familiarity, a general sense of oldness without detailed information (Long and Prat, 2002; Herzmann and Curran, 2011). This study asks how expertise affects associative memory and recognition for semantic information that is presented incidentally.

This study was also motivated by a question of whether the expertise advantage could transfer to information outside of the realm of participants’ expertise, provided that an associative link is learned between the unrelated information and expertise-related information. This question has not, to our knowledge, been reported in the expertise literature, other than a study that showed an expertise advantage in older adult accountants (number experts) for arbitrarily assigned numbers given a learned associated location (Castel, 2007). Here, we specifically investigate if this transfer can occur between expertise-related words and expertise-unrelated images. We chose this association between images and words because it is a particularly unique type of association, which requires forming connections between verbal and non-verbal representations that are processed differently (e.g., Tversky, 1969). The dual-coding theory, for example, refers to these two functionally and structurally distinct systems, one verbal and one non-verbal, as well as, importantly, an integrative mechanism that connects the systems (Paivio, 1986; Paivio, 2007). This integrative mechanism allows for an image to act as a cue for remembering an associated word. Our investigation probes the question of whether the integrative mechanism between two separate systems is influenced by expertise.

Although it is true that very few studies have compared experts and non-experts in their abilities to form associative memories, the sports psychology literature is an exception to this. The focus of these studies, however, is typically on procedural learning, as opposed to semantic learning. One study, for example, investigated the role of image–word associations in connecting mental imagery with internal verbalizing during procedural learning (Schack et al., 2014). Our study complements these past associative memory studies of expertise by investigating the unique relationship between images and words when advancing semantic learning.

To summarize, we examine how expertise supports forming associations between incidentally presented expertise-related and arbitrarily related pieces of knowledge, with a focus on words within the bounds of the expertise, and unknown images (in this case, unknown faces) that lie outside the bounds of the expertise. To do this, we exposed people who have relatively greater, or relatively less, expertise in sports knowledge (“sports experts” versus “non-sports-experts”) to unknown faces in an incidental encoding paradigm where participants answered simple perceptual questions about facial features. Each face was presented with a label (“descriptor”) as belonging to either a prospective athlete for a sports team (within the domain of expertise for the sports-experts only; e.g., sport-experts are more familiar with the role of a punter), or a hypothetical new employee for a (sports-unrelated) business (within the domain of expertise of both groups; e.g., both groups understand the role of a waiter). Critically, the descriptor was incidental and completely irrelevant to the perceptual task. We later gave participants surprise memory tests of their: recognition memory of the faces, ability to recall associated information from when they studied the faces, and ability to select the correct descriptor for each associated face, as well as confidence in their selections. We hypothesized that experts would be able to use their relatively extensive semantic memory to more rapidly form connections in memory with incidentally encountered information within their subject area, allowing them to be more successful than non-experts at retrieving the descriptors associated with faces, and at matching faces with the correct descriptor, for the prospective athletes compared to non-sports-experts. However, there should be no difference as a function of sports expertise for the prospective business employees. Based on the reviewed findings of intentional encoding, we further predicted that the expertise advantage should be specific to remembered details, whereas overall recognition memory should be relatively similar between experts and non-sports-experts.

## Materials and Methods

### Participants

One hundred and thirty participants were recruited for this study. We aimed to have 30 participants per group at the analysis stage to match the sample sizes used in similar previous studies (e.g., Long and Prat, 2002; Rohrmeier and Widdess, 2017). Results from 20 pilot subjects indicated that performance on a sport knowledge test could be used to create our initial three groups, where the top one-third of performers would represent experts, the bottom one-third of performers would represent non-sports-experts, and middle performers would not be analyzed further. To account for drop-out rates, we included a buffer of 40 participants (about 13 buffer participants per group). After exclusion criteria were considered (see “Participant Expertise”), 38 sports-experts and 34 non-sports-experts were included in the final analyses.

In order to access a diverse group of sports experts that would have been inaccessible through local recruitment, we recruited participants through Amazon’s Mechanical Turk (MTurk ^1^), a large online participant pool. Prior work has shown that this tool produces similar levels of reliability as in-person laboratory studies (Buhrmester et al., 2011). The inclusion criteria for the study required participants to (1) have at least 95% MTurk approval ratings and have completed over 100 prior MTurk tasks, (2) have always lived in the United States, (3) have no identified cognitive disability, (4) be native English speakers, and (5) be older than 18 years of age. The study was approved by the University of Pittsburgh Institutional Review Board and all participants provided online informed consent prior to data collection.

### Stimuli

Twenty athlete and 20 non-athlete “descriptors” were created. In the athlete condition, these descriptors were randomized combinations of 20 unique professional sports teams and 20 unique sports positions; these consisted of 10 combinations of team names and positions (e.g., Steelers punter) from American football, seven from baseball, and three from basketball. Each team was from a different state or the District of Columbia in the United States, and teams were distributed evenly across the country. For the non-athlete condition, descriptors were pairs of 20 unique jobs and states (e.g., Colorado doctor). The states used in the non-athlete condition were the same states that host each of the sports teams in the athlete condition, to avoid differences related to geographic proximity.

**FIGURE 1:**
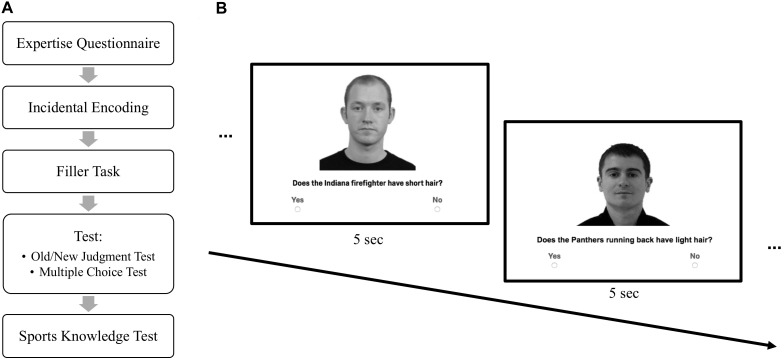
Experimental Methods. **(A)** Order of task presentation and **(B)** incidental encoding phase. Face images presented were acquired from online databases (Langner et al., 2010; Tarrés and Rama, “GTAV Face Database”). Each database obtained permission from models for future publication of images in scientific journals.

Eighty color face images were chosen from face databases (Langner et al., 2010; Tarrés and Rama) and online searches. Images consisted of White or Moroccan Dutch men, facing forward with neutral expressions, wearing black t-shirts on a white background. Images contained the individual’s face, hair, and neck (see Figure 1B). None of the individuals in the images were actual professional athletes or otherwise recognizable. The images were separated into two sets (40 in each set): one used in the learning phase and the other used as “new” images in the old/new judgment task.

For each image used in the incidental encoding phase, two questions were created that asked about facial features (e.g., eyebrows). A descriptor was embedded in each question based on the randomized assignment of images to the athlete/non-athlete conditions (e.g., Does the Steeler punter/Colorado doctor have thick eyebrows?). The questions about facial features served purely as a vehicle for incidentally delivering the descriptor information – participants did not need to remember these particular facial features for any subsequent memory tests. The athlete vs. non-athlete assignment was counterbalanced across participants so that any condition differences could not be due to systematic differences in the faces and/or questions presented. Both presentations of each face had the same descriptor, but different questions about their facial features (e.g., Does the Steelers punter have dark hair?; Does the Steelers punter have a beard?). The correct answer was always “yes” to one and “no” to the other. The order of “yes” and “no” response questions was counterbalanced.

### Procedure

Participants first completed a questionnaire that assessed their perceived sports knowledge. Next, they participated in the incidental encoding phase, followed immediately by a filler task. Finally, participants completed a surprise memory test and a sports knowledge test (see Figure 1A).

#### Sports Knowledge Assessment

We categorized participants according to their relative expertise with sports knowledge, based on their performance on a sports knowledge test (conducted at the end of the study to avoid perceived success from biasing subsequent responses), and confirmed this categorization through participants’ judgments of their own knowledge. Expertise has been measured in a variety of ways, though for our examination of new semantic learning (associations between faces and descriptors), we measured expertise in terms of semantic knowledge. Several examples of this approach to assessing expertise include studies of how the brain responds to images in a domain of expertise. In James and James (2013), a test of 37 questions was administered to evaluate Pokémon expertise, and in Grelotti et al. (2005), perceptual expertise was determined through a force-choice verification task requiring knowledge of the names of Digimon characters. Our assessments of semantic knowledge (e.g., “What position was played by Jerry Rice?”) is consistent with these other expertise studies.

##### Self-report sports expertise questionnaire

Participants rated their perceived level of sports expertise by indicating their level of agreement with four statements using a 7-point Likert scale. Statements regarding their knowledge of sports in general, and within the three popular sports in the United States: football, baseball, and basketball were used (e.g., “I feel competent about my knowledge in sports”; “Compared to others, I know less about sports”). Such questions have been used in the past to help classify participants’ relative expertise (Perrouty et al., 2006). The perceived expertise statements were followed by four additional questions: whether participants followed sports media, whether they played sports, and how frequently they engaged in either watching sports media and/or playing sports.

##### Sports knowledge test

As an objective assessment of sports expertise, participants had four minutes to answer 15 four-option multiple-choice questions about football, baseball, and basketball (five questions each). Questions focused on each sport’s rules, famous players, and professional teams (e.g., What position was played by Jerry Rice?). Participants were instructed not to look up answers online. Beyond the implementation of the time constraint, there is evidence that participants did rely on their own knowledge to complete the test. Mainly, to anticipate the results, we found that expertise group differences on test performance corresponded to equivalent differences in self-perceived expertise (which was probed at the start of the study when participants had no knowledge that they would later be tested). Following the knowledge test, demographic information, including age, sex, highest education level, industry of work, current job title, and the states lived in for over a year, were collected.

#### Incidental Encoding Phase

Participants were informed that they would be shown a series of face images and were asked to imagine that some of the men in the images had been recently drafted for professional sports teams or hired for various jobs across the United States. The participants’ task was to answer yes/no questions about the physical features of the men in the images (see Figure 1B). Each of the 40 images (20 athletes, 20 non-athletes) was presented twice with a different question each time (as described above). In order to minimize distractions and maintain participants’ attention on the task, the response time for each trial was limited to 5 s, with the next trial beginning following 5 s, regardless of participants’ responses.

#### Filler Task

Following the incidental encoding phase, participants performed an English vocabulary test for 5 min to clear their working memory.

#### Memory Tests

##### Old/new judgment test

Participants were presented with one facial image at a time and asked if it was previously presented. If they responded “yes,” they were asked to “Please type what you remember about the person below (e.g., job, position, state, team)” into a single-line text box. If the participant reported “no,” the next image was presented. Half of the images from the encoding phase were used for the judgment test (i.e., 10 “old” images from each condition), along with 20 “new” previously unseen facial images. This test allowed us to investigate participants’ recognition of the images with the first question. Prompting participants to type in remembered details then allowed us to measure the recollection of the associative memories they had formed.

##### Multiple-choice test

On each trial, three images that had previously appeared in the encoding phase were presented along with a descriptor (e.g., the team and position labels) that had been previously associated with one of the images. Participants were asked to pick the image that matched the descriptor from three choices: the target face, a face from the same condition (e.g., another athlete), and a face from the other condition (e.g., a non-athlete). After the participant responded to each question, they were asked to rate their confidence on a 4-point scale. Items were presented in a random order and images on the screen were in random positions. This test allowed for measuring associated memory for the face-descriptor pairs. This test again relies on recollection during retrieval because a sense of oldness (familiarity) was not sufficient to get the answer correct when choosing from faces that were all old.

### Data Analysis

Based on accuracy in the sports knowledge test, the top one-third of participants were classified as experts, and the bottom one-third were classified as non-sports-experts. Performance during the incidental encoding phase was defined by participants’ accuracies at judging the physical features (e.g., whether the individual has a beard), and ratio of null responses. This performance was compared between participants and descriptors to ensure there were no differences in participants’ behavior during encoding.

Two outcome variables were calculated from responses to the old/new judgment and multiple-choice tests. Based on the responses to the old/new judgment test, *d′* (to avoid response biases) was calculated using the hit rate and false alarm for each of the athlete and non-athlete descriptors. For each hit trial, we further calculated the number of correctly recalled descriptor details (range: 0–2) to indicate the quantity and quality of recalled information (team and position for athletes, job, and state for non-athletes). Participants’ cued-recall performance was defined as the average number of recalled details over hit trials. For example, consider a participant who was correct for five faces being old. If the participant recalled four separate pieces of information about those five faces [e.g., Player 1: remembering they played for the Steelers but not their position (1); Player 2: remembering they were a pitcher but not the team (1); Player 3: remembering they were point guard for the Nicks (2); Players 4 and 5: remembering nothing (0)], their sports recall score would be the number of recalled pieces of information (4) divided by hit trials (5), giving a score of 0.80.

For the multiple-choice test, accuracy was calculated as the percentage of correctly identified descriptors for each face. Confidence was calculated as the average confidence rating from the 4-point scale on trials where participants correctly identified the target. Each of the learning and test, measurements was analyzed with 2 (Expertise: sports-expert, non-sports-expert) × 2 (Descriptor: athlete, non-athlete) mixed ANOVAs. Additionally, covariates were determined by evaluating if the expertise groups differed on age, education level, or number of states lived in using independent *t*-tests, and on gender using a chi-square. Variables reaching significance of *p* < 0.05 were included as covariates in 2 (Expertise: sports-expert, non-sports-expert) × 2 (Descriptor: athlete, non-athlete) mixed ANCOVAs. Following Schneider et al. (2015), any covariate and within^∗^covariate interactions are reported from the ANCOVA results; all other *F* test results reported are from the ANOVA (Schneider et al., 2015). Paired *t*-tests were conducted for all significant interactions to examine the effect of expertise on the relevant outcome variable.

## Results

### Participant Expertise

Based on accuracy on the sports knowledge test, 44 sports-experts (performance: *M* = 0.80, *SD* = 0.10) and 47 non-sports-experts (performance: *M* = 0.25, *SD* = 0.10) were identified based on their accuracy on sports knowledge test for a total sample of 91 participants. Data from five experts and 13 non-sport-experts were excluded from analyses due to low performance in the incidental encoding phase (null response in more than 10% of trials or overall accuracy below 70%). One additional expert was excluded because of extremely poor performance in the old/new judgment task, suggesting they did not complete the task appropriately (*d′* < 0). As a result, 38 sports-experts and 34 non-sports-experts were included in the final analyses. Table 1 reports descriptive statistics. As expected based on how we defined our groups, sports-experts performed significantly better in the sport knowledge test than non-sports-experts, *t*(70) = 23.65, *p* < 0.001, *d* = 5.60. Scores from the participants’ self-evaluation of sport expertise further confirmed the group difference, *t*(70) = 6.33, *p* < 0.001, *d* = 1.52. The two groups were comparable in age, education level, and the number of states lived in (all *p*s > 0.31). However, there were more males than females in the expert group than the non-sports-expert group, χ^2^(1, *N* = 72) = 24.40, *p* < 0.001, Φ = 0.58. Therefore, gender was included as a covariate for all further analyses. All ANOVA and *t*-test results are reported below. Only significant ANCOVA results are reported below (other *F*s < 1).

**Table 1:** Descriptive statistics of sports experts and non-sports-experts.

	Sports-Experts	Non-sports-Experts
**Descriptive statistics**		
*N*	38	34
Gender (*N*)		
Male	32	6
Female	6	28
Age	40.8 (12.3)	38.6 (10.8)
Education level completed (*N*)		
High school or equivalent	4	5
Vocational/technical school (2 years)	1	0
Some college	8	13
College graduate (4 years)	18	12
More than 4 years college	7	4
Number of states lived in for over a year	2.0 (1.8)	1.7 (1.3)
**Sports knowledge assessments**		
Sports knowledge test performance	0.80 (0.10)	0.25 (0.10)
Self-report sports expertise	4.67 (0.94)	3.56 (0.44)
**Incidental encoding task performance**		
Null response rate		
Athlete	1.32 (1.09)	0.97 (0.97)
Non-athlete	0.66 (0.82)	0.97 (1.09)
Accuracy		
Athlete	0.86 (0.06)	0.85 (0.08)
Non-athlete	0.91 (0.05)	0.88 (0.05)

*Means reported with standard deviations in parentheses unless otherwise indicated.*

### Incidental Encoding Phase

Participants performed well in the (incidental) perceptual task that was administered during encoding, demonstrating that they completed the task attentively (see Table 1). Incidental task performance did not differ between sports-experts and non-sports-experts or between athlete and non-athlete descriptors, and there was no interaction of the variables for null response rates (all *p*s > 0.144) or task accuracy (all *p*s > 0.335). This suggests that any subsequent significant differences cannot be attributed to differing levels of engagement in the task.

### Old/New Judgment Test

**FIGURE 2:**
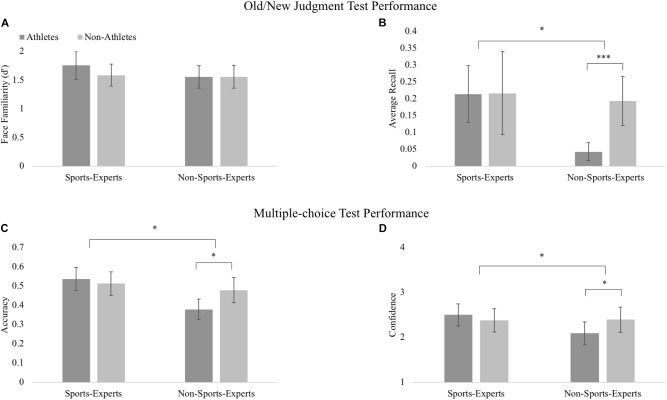
Memory test performance. Performance on: **(A)** Old/New test (*d′*); **(B)** recall of descriptor details (see Data Analysis for full calculation of *y*-axis); **(C)** multiple-choice accuracy for matching face-descriptor pairs when presented along with two old lures; **(D)** multiple-choice confidence. Error bars reflect 95% confidence intervals. Significance is indicated as follows: ^∗∗∗^*p* < 0.001; ^∗^*p* < 0.05.

For old/new recognition judgments, when participants judged whether each face was old or new, we compared *d′* values based on the hit rate and false alarm for each of the athlete and non-athlete descriptors. Sports-experts and non-sports-experts were comparable (*F* < 1), as were athlete and non-athlete descriptors, *F*(1, 70) = 1.64, *p* = 0.205, η_p_^2^ = 0.023. There was no interaction between group and descriptor, *F*(1, 70) = 2.00, *p* = 0.162, η_p_^2^ = 0.028 (see Figure 2A).

For the number of details recalled about familiar faces, an expertise advantage was present: a significant interaction was found between group and descriptor, *F*(1, 70) = 5.571, *p* = 0.021, η_p_^2^ = 0.074. Specifically, non-experts remembered more details about non-athletes than athletes, *t*(70) = 4.023, *p* < 0.001, *d* = 0.92. In contrast, experts recalled comparable details for non-athletes and athletes, *t*(70) = 0.048, *p* = 0.962, *d* = 0.01 (see Figure 2B). Collapsing the groups showed people recalled more details about non-athletes than athletes (for trials correctly judged as old), *F*(1, 70) = 5.933, *p* = 0.017, η_p_^2^ = 0.078. Collapsing the types of images (athlete/non-athlete) led to comparable performance in experts and non-sports-experts, *F*(1, 70) = 3.236, *p* = 0.076, η_p_^2^ = 0.044.

### Multiple-Choice Test

We compared participants’ accuracies at choosing the correct face given a descriptor among two old lures. Sports-experts had greater overall multiple-choice accuracy than non-sports-experts, *F*(1, 70) = 7.27, *p* = 0.009, η_p_^2^ = 0.094. The interaction between expertise and descriptor was also significant, *F*(1, 70) = 6.14, *p* = 0.016, η_p_^2^ = 0.081. This was driven by non-sports-experts having higher accuracy when associating descriptors to non-athlete faces than when associating descriptors to athlete faces, *t*(70) = 2.42, *p* = 0.021, *d* = 0.56 (Figure 2C). The two descriptors were comparable for experts, *t*(70) = -0.808, *p* = 0.424, *d* = 0.13, consistent with the expertise advantage. Collapsing across expertise, there were no differences between athlete and non-athlete images, *F*(1, 70) = 2.34, *p* = 0.131, η_p_^2^ = 0.032.

Experts and non-sports-experts had similar overall confidence levels [*F*(1, 70) = 1.31, *p* = 0.256, η_p_^2^ = 0.018], and participants were similarly confident about their responses to non-athletes and athletes, *F*(1, 70) = 1.66, *p* = 0.202, η_p_^2^ = 0.023. There was a significant interaction of confidence between descriptor and expertise, *F*(1, 70) = 9.17 *p* = 0.003, η_p_^2^ = 0.166. While sports-experts had similar confidence when associating non-athletes and athletes [*t*(70) = -1.20, *p* = 0.238, *d* = 0.155], non-sports-experts were more confident when associating non-athletes than athletes, *t*(70) = 3.18, *p* = 0.003, *d* = 0.39 (see Figure 2D). There was a significant interaction between descriptor and the gender covariate, *F*(1, 70) = 4.19, *p* = 0.045, η_p_^2^ = 0.06, although this relationship was not significant when examined outside the full model (*p*s > 0.72).

## Discussion

In this study, we report an expertise advantage for incidentally learned word–image associations for words within a person’s domain of expertise, and unknown arbitrary images. Sports-knowledge experts showed superior memory for linking incidentally encountered expertise information (team and position) with faces, compared to non-sports-experts. In contrast, sports-experts and non-sports-experts did not show differences in memory performance for a shared topic of expertise (jobs in businesses).

While previous studies have shown an expertise advantage in learning domain-relevant information (Chase and Simon, 1973; Deakin and Allard, 1991; Long and Prat, 2002), our findings indicate that an expertise advantage can be expanded to include images that are not a focus of the expertise (i.e., arbitrary unfamiliar faces). This advantage emerged even when the information was incidentally embedded within a basic perceptual question that linked expertise-relevant information with the domain-unrelated images (faces). Though incidental and intentional types of encoding differ in various respects, our findings suggest that, like intentional encoding, incidental encoding receives an advantage from encountering expertise-relevant knowledge. Our finding of these effects for associations between images and words demonstrate that the expertise advantage is unaffected by the integrative mechanism between verbal and non-verbal systems (Paivio, 1986).

The expertise advantage we observed might be explained by experts’ more extensive and organized related knowledge, allowing for automatically deeper and more parsimonious encoding of unknown expertise-domain information and associated images, compared to non-experts. Previous research has indicated that new word-image associations can be rapidly integrated into memory when this unknown information is linked with existing knowledge (McClelland, 2013; Coutanche and Thompson-Schill, 2015). Here, we propose that sports-related information (incidentally retrieved in the perceptual task) activated the experts’ semantic domain-specific knowledge, allowing for deeper and more rapid encoding of the information compared to non-sports-experts, who do not have this same organized prior knowledge. In contrast, both sports-experts and non-sports-experts have domain knowledge concerning the role of common jobs in businesses, allowing both groups to similarly benefit from deep rapid encoding of information for these word-image associations. An interesting question beyond the scope of this study is at what point during the memory process these differences emerge. We do not believe that an attentional effect can explain our results (discussed further below), but the memory process itself involves a range of processes from recognition through to deep encoding. Although the differences we observed might be driven by encoding differences between experts and non-experts, it is also possible that the differences begin at a later stage of memory, such as maintenance or perhaps being better at recalling expertise-relevant information. Conducting a similar study while measuring neural activity with a temporally sensitive method, such as electroencephalography (EEG), might prove useful for probing precisely when these expertise-driven differences occur (Herzmann and Curran, 2011).

Our findings showed an expertise-advantage for associative memory, but not recognition of individual faces. Though incidental and intentional encoding differ in various respects, our findings suggest that the expertise-advantage present for intentionally encoded associations (Long and Prat, 2002) is also seen for incidentally learned associations between words and images, even when the images themselves are not part of the domain of expertise (i.e., faces, rather than team logos). Dissociations between intentional and incidental encoding in other areas of memory (e.g., Sharon et al., 2011) illustrate that this was not an inevitable finding, and our results add to our knowledge of domains in which intentional and incidental encoding show similar versus dissimilar effects.

What is required for a person to be an “expert”? The criteria for “expertise” are often relative, based on to whom potential experts are compared. For example, a Ph.D. student might be considered an expert in their area by someone outside that field, but not by a Postdoctoral Fellow or faculty member. Our definition of expertise is similarly relative, as we compare participants with greater knowledge of sports to those with less knowledge. There are no doubt greater experts in sports knowledge in the population, but we consider our findings as reflecting the effects of *relatively* greater expertise. Our assessments of this expertise – a knowledge test and self-perception – align with prior studies that have considered participants “experts” based on their knowledge of Pokémon (Grelotti et al., 2005) and Digimon (James and James, 2013) characters, or based on their self-reports of their knowledge (Perrouty et al., 2006). We note that our expertise criteria required that participants show expertise in more than one of the included sports, meaning it was possible to have a baseball expert will no knowledge of basketball or football in our non-sports-expert group, for example. Although this means that the non-sports-expert group is not necessarily ignorant of all sports, it has the consequence of our sports-expert group having expertise across sports, rather than in a single sport. This does not, however, affect the validity of the found effects, as the presence of expertise for one sport in the non-sports-expert group would only weaken the distinction we observed.

How can we be certain that participants were learning incidentally and not intentionally? This is a common concern for the field of incidental encoding. In our study, we employed a cover task during encoding (making judgments about perceptual features of faces) and did not warn participants that memory tests were forthcoming. Participants’ performance in the multiple-choice and recall tests was consistent with incidental encoding, which typically gives lower memory performance than does intentional learning. For example, the 3-way alternate forced choice performance was below 60% (chance = 33.3%) for all descriptors and expertise groups (Figure 2C). Compare this to *a prior* study (Experiment 1 in Coutanche and Thompson-Schill, 2014), in which a similar three-way alternate forced choice test gave a mean of 80.7% after intentional encoding and 56.2% after incidental encoding. One interesting question for future research is examining the strategy that participants might be using. One possible strategy in this study would be naming the faces, thereby creating labels for them (though without warning that a memory test is forthcoming, this would need to be a spontaneous approach taken while making perceptual judgments). If this were the case, the strategy would likely have occurred in both descriptors, so would not explain our key effects. In this study, in which images and words were presented, one relevant individual difference could be the extent to which a person is characterized a “visualizer” or “verbalizer” (Alfred and Kraemer, 2017). Individual differences can modulate the extent to which prior knowledge aids new learning (e.g., Coutanche and Koch, 2017), raising the intriguing possibility that individual differences in how new material is processed might interact with the effects of expertise.

Because items that are better attended are often better remembered (Chun and Turk-Browne, 2007), one concern may be that the observed expertise advantage could be explained by experts paying more attention to athlete faces than to non-athlete faces. Performance on the incidental encoding task, however, is inconsistent with this concern – the two groups showed similar performance, suggesting similar levels of attention. Further, the two groups had comparable performance on the old/new judgment task. If attention were playing a strong role, we would have expected sports-experts to show superior performances for athlete faces, relative to non-sports-experts in this task. We also note that participants’ gender was not balanced (with more males in the sports-experts group). However, based on a recent meta-analysis, females typically outperform males on recognizing male faces (Herlitz and Lovén, 2013), which – if gender was playing a role – would predict better memory of faces for the non-sports-experts group (mainly females) than sports-experts (mainly males) overall, which we did not find. Furthermore, a gender difference in face memory would not explain the interactions we found between group and descriptor in associative memory.

Despite the benefits of using MTurk to collect participant data, this approach also has limitations. As with all research collected outside a lab, it can be difficult to guarantee with certainty that participants give complete focus to a task, or follow instructions (i.e., be native English speakers; refrain from checking answers with a friend). We do not believe this affected our own results, as performance during the incidental task was strong, and we report a within-participant interaction [so that any non-task distraction would need to disproportionately occur for half the (interleaved) trials, for one group only]. Our selection of highly rated MTurk users helps guard against further deception, though this is always a possibility. Another limitation is that the generalizability of the sample was limited, as we only collected data from those with active MTurk accounts, though the sample from MTurk has been shown to be more demographically diverse than typical college samples (Buhrmester et al., 2011).

To summarize, we have found that expertise boosts the encoding of associations between expertise-relevant words and arbitrary images, even when these pairings are incidentally encountered. An expertise-advantage was not observed for face recognition. Future studies might consider which other types of associated information can receive a benefit from expertise.

## Author Contributions

XF, DK, EH, and MC conceived and developed the study. HB collected the data with assistance from XF, DK, and EH. HB, MC, XF, and DK contributed to data analysis. HB drafted the manuscript and received feedback from all authors. All authors contributed to the study design and approved the final version of the manuscript for submission.

## Conflict of Interest Statement

The authors declare that the research was conducted in the absence of any commercial or financial relationships that could be construed as a potential conflict of interest.

## Footnotes

1 www.mturk.com
